# Extranodal Involvement of the Anorectal Region in an HIV-Positive Patient With Plasmablastic Lymphoma: A Case Report

**DOI:** 10.7759/cureus.33151

**Published:** 2022-12-30

**Authors:** Tanmay Gandhi, Aniruddh Shah, Aishwarya Thakurdesai

**Affiliations:** 1 Department of Internal Medicine, University of Arkansas for Medical Sciences, Little Rock, USA; 2 Department of Internal Medicine, Cleveland Clinic Foundation, Cleveland, USA; 3 Department of Internal Medicine, University of Louisville, Louisville, USA

**Keywords:** b cell lymphoma, hiv-positive, extranodal, anorectal, plasmablastic lymphoma

## Abstract

Plasmablastic lymphoma (PbL) is a rare type of aggressive B-cell malignancy that has an extremely poor prognosis without chemotherapeutic treatment, requiring a high degree of suspicion for an early and accurate diagnosis. It has been classically described in patients infected with the human immunodeficiency virus (HIV). However, it accounts for only 2.6% of acquired immunodeficiency syndrome (AIDS)-related lymphomas. Extranodal involvement is most commonly seen within the oral cavity (44%). Involvement of the gastrointestinal tract (14%) is rare and can often be confused with other malignancies with plasmablastic features. We present a rare case of PbL in a 55-year-old male with HIV-AIDS (CD4 (cluster of differentiation 4) cell count of 128), who presented for evaluation of incidentally detected multiple liver masses and lytic lesions in the ribs. Further workup revealed evidence of a lesion with increased uptake in the anorectal region with fine needle aspiration (FNA) biopsy identifying the lesion as plasmablastic lymphoma.

## Introduction

Plasmablastic lymphoma (PbL) falls under the broad category of diffuse large B-cell lymphoma (DLBCL). This entity was first described in 1997 in a group of 16 patients (15/16 patients being human immunodeficiency virus (HIV) positive), with lesions primarily involving the oral cavity [1]. It has since been classically associated with HIV-positive patients [2]. However, despite the high degree of association with HIV, it accounts for only 2.6% of acquired immunodeficiency syndrome (AIDS)-related lymphoma [3], making it a relatively rare diagnosis. Among these HIV-positive patients with PbL, the involvement of the gastrointestinal (GI) tract is rare (12%), with the majority of the extranodal lesions being in the oral cavity (48%). We hereby describe a case of PbL with extranodal involvement of the anorectal region in this patient with HIV AIDS.

## Case presentation

A 55-year-old male, with untreated HIV, presented for evaluation of newly diagnosed plasma cell malignancy. Past medical history was significant for HIV (cluster of differentiation 4 (CD4) cell count at admission - 124), heart failure with reduced ejection fraction (HFrEF) status post implantable cardioverter defibrillator (ICD) placement, atrial fibrillation, and chronic obstructive pulmonary disease (COPD) on 2 liters of oxygen at home.

The patient presented one week ago to an outside hospital with complaints of progressively worsening acute onset of shortness of breath. He had a computed tomography (CT) scan of the chest and abdomen revealing pneumonia, multiple lytic lesions in the ribs, and multiple liver masses. A liver biopsy was performed revealing a plasmacytic infiltrate with an expression of Ki-67 > 90%. The cells were positive for CD138, CD56, Epstein Barr Virus (EBV)-encoded RNA (EBER) and negative for CD3, epithelial membrane antigen (EMA), and CD45. The plasma cell infiltrate was shown to demonstrate kappa clonality. Subsequently, the patient was transferred to our institute for further management and care.

The workup performed at our hospital included - a bone survey that did not reveal any evidence of lytic lesions, a bone marrow biopsy revealing no evidence of multiple myeloma, positron emission tomography (PET)-CT, which showed multiple, low-density, hypermetabolic hepatic masses, and multifocal lytic expansile lesions throughout the axial and appendicular skeleton. Interestingly, there was increased radiotracer uptake within the distal rectum near the anal and rectal junction.

The findings of the colonic lesions on the PET-CT were evaluated for underlying malignancy through a colonoscopy, which revealed a submucosal lesion of 1.5 cm x 1.5 cm, 3 cm from the anal verge with normal overlying mucosa. Subsequent endoscopic ultrasound (EUS) revealed an oval, hypoechoic, and heterogenous mass measuring 3.2 cm x 2.7 cm. The wall of the mass was well-demarcated and smooth. The mass appeared to arise from the muscularis layer (endoscopic ultrasound (EUS) layer 4) raising suspicion for leiomyoma, with an intact muscularis noted along the entire lesion. There was no evidence of adjacent lymphadenopathy.

**Figure 1:**
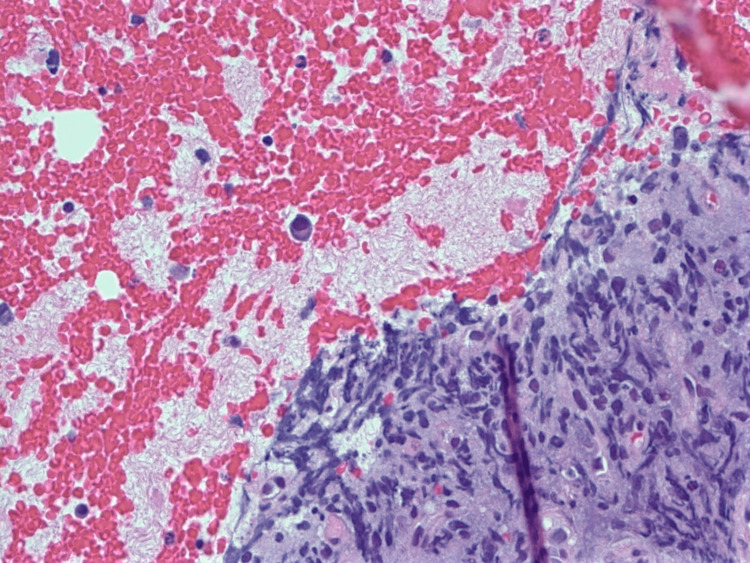
FNA biopsy, H&E stain Some crush artifacts within an aggregate of neoplastic cells. A large cell is seen within the background of blood, which appears to be an atypical plasmacytoid cell. FNA: fine-needle aspiration; H&E: hematoxylin and eosin

**Figure 2:**
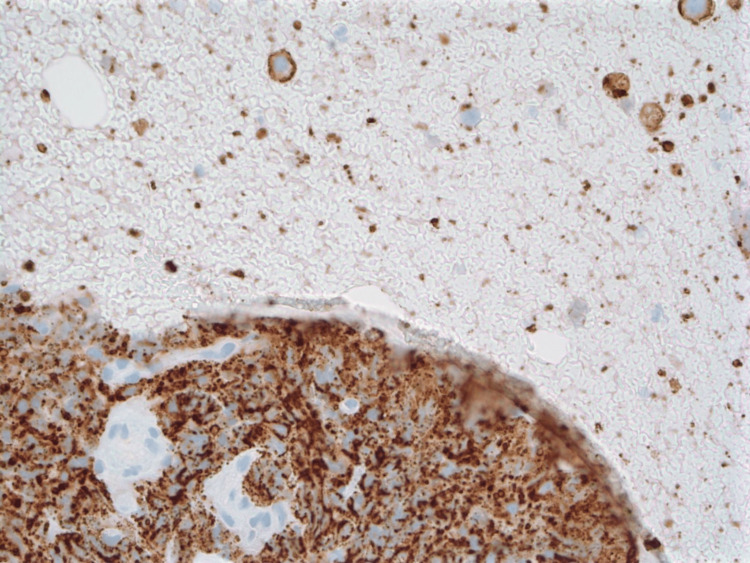
CD138 stain Positive cluster of differentiation (CD) 138 staining (seen as brown chromogen) of the cluster of crushed cells and isolated plasmacytoid cells

**Figure 3:**
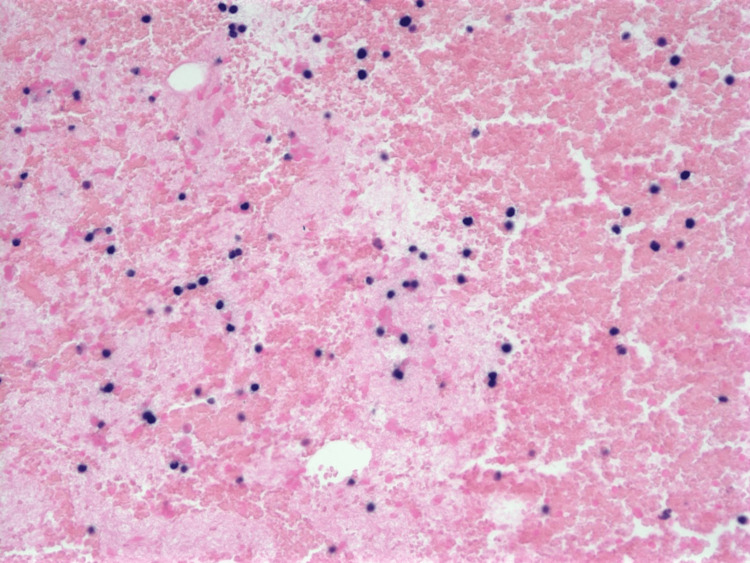
EBER stain Positive EBER (Epstein Barr Virus (EBV) in situ hybridization) staining (seen as dark purple) in many cells

FNA of the lesion revealed scattered and focal clusters of plasma cells with some crush artifacts (Figure 1). The plasma cells were positive for CD138 (Figure 2) and negative for pan-Cytokeratin (pan-CK). The EBV in situ hybridization (ISH) stain was positive (Figure 3). 

Flow cytometric analysis showed cells positive for CD138, CD38 (dim/heterogenous), CD56, and CD81; they were negative for CD45, CD19, and CD20. Kappa and lambda light chain analysis showed kappa restriction. Fluorescence in situ hybridization (FISH) revealed gene rearrangement involving 8q24 (c-MYC) and a gain on 14q32. Human herpes virus (HHV)-8 by polymerase chain reaction (PCR) was negative. At this point, a diagnosis of plasmablastic lymphoma was made.

At the time of hospitalization, the patient was not taking any anti-retroviral medications. His CD4 count was 124. He was started on antiretroviral treatment with emtricitabine, tenofovir, and dolutegravir. He was found to have EBV viremia with 26,000 copies, which progressively increased to 381,000 at the time of discharge. This finding was discussed with the infectious disease team, which determined that no treatment was necessary.

The patient was started on bortezomib, cyclophosphamide, doxorubicin hydrochloride, vincristine sulfate, and prednisone (bortezomib-CHOP) chemotherapy with good tolerance. He was also considered for daratumumab therapy, as his lymphoma cells were CD38 positive. However, due to insurance-related barriers, there was a delay in adding daratumumab therapy, which was ultimately started later with his fourth cycle of bortezomib-CHOP.

Continual follow-up of the patient revealed that the patient had achieved complete remission about five months after initiation of treatment. He remained in remission for five months after which he was found to have a relapse involving a mass in the chest located between the aorta and pulmonary vein. A biopsy was performed, which confirmed the relapse. He was subsequently started on salvage therapy with bortezomib, dexamethasone, cisplatin, cyclophosphamide, and etoposide. Adriamycin was not used due to HFrEF with an ejection fraction of 20%. Follow-up PET-CT after the first cycle of salvage chemotherapy showed considerable response with complete resolution of his chest mass. He was planned for a total of six cycles of chemotherapy; however, at his fifth cycle and 10 months after his initial relapse, his repeat PET-CT showed a relapse of the disease at the same site as before. Due to the poor functional status, the patient was not considered a candidate for further chemotherapy and was transitioned to comfort measures through palliative care management.

## Discussion

HIV and AIDS have been known to be associated with a number of lymphoproliferative disorders [4]. Among these, non-Hodgkin lymphomas (NHLs) contribute to a majority of AIDS-defining malignancies. Most of these NHLs arise systemically with extranodal involvement such as that of the gastrointestinal tract. The most common histologic subtypes of AIDS-NHL include Burkitt’s lymphoma and DLBCL with other rare subtypes including primary effusion lymphoma, anaplastic lymphoma kinase (ALK)-positive DLBCL, plasmablastic lymphoma, and HHV-8-related DLBCL [5].

PbL is classically described among patients with HIV, with a predominance among males [2]. However, over the years, it is known to be seen in immunocompetent and organ transplant patients as well [2]. The median age of diagnosis is around 50 years, with an earlier age of onset seen among the HIV-positive subgroup [3,4]. Most cases present in an advanced stage of the illness, Ann Arbor stage 3 or 4; however, this Ann Arbor staging in cases of PbL has not shown any prognostic correlation [2,4]. The diagnosis of PbL carries a guarded prognosis with a median survival time of 6-19 months with no clear difference among the HIV-positive and HIV-negative populations. The most common presentation is extra-nodal involvement, with the most common site being the oral cavity (44%), followed by the gastrointestinal (14%), and skin (7%) [6]. However, it is interesting to note that patients with HIV-negative PbL showed a higher gastrointestinal involvement (21%) as compared to HIV-positive PbL (12%) [6]. They also showed lower rates of oral involvement (40% versus 48%) and lower rates of EBV positivity (46% versus 82%) as compared to those with HIV-positive PbL [7,8]. HIV-negative PbL also presented with B symptoms and frequent bone marrow involvement [9].

While gastrointestinal (GI) involvement in PbL is seen in 14 % of cases, the diagnosis can be challenging due to the morphologic similarities with other malignancies with plasmablastic features such as Burkitt’s lymphoma, plasmablastic plasma cell myeloma, other types of CD20-negative DLBCL (such as primary effusion lymphoma, ALK-positive DLBCL) and other HHV-8-positive large B-cell lymphoma [6,10,11]. PbL differs from DLBCL in that it is negative for immunophenotypic markers such as CD20 and CD45 [1]. While PbL closely matches the immunophenotype of a plasmablastic plasma cell myeloma, there is the absence of significant bone marrow involvement, a monoclonal protein identified in the serum, and close association of PbL with EBV [12] versus a plasmablastic plasma cell myeloma, which is found to carry the virus in only 6.7% of the cases [13].

Hence, the accurate identification and diagnosis of a PbL require a thorough immunophenotypic workup.

The classic immunophenotypic markers seen in PbL include positivity for CD38, CD138, MYC, IRF, and MUM1 with variable expression of CD45, CD30, EMA, and CD79a [3]. There is, usually an absence of the pan B-cell markers of CD20 and PAX 5 [3]. They also usually have a Ki-67 index > 90% demonstrating hyperproliferation [14].

Comparative studies have shown no differences in survival among HIV-positive PbL and HIV-negative PbL [2]. However, those among HIV-negative PbL who are immunosuppressed have been shown to have significantly poor median survival (six months) as opposed to those without immunosuppression (13 months) [7]. The use of highly active antiretroviral therapy (HAART) therapy in patients with HIV-positive PbL has shown better survival in some studies; however, other studies have shown no difference [6,8]. Patients with MYC gene rearrangements have shown poor outcomes and shorter overall survival [9]. The impact of EBV positivity and viral load on outcomes is unclear, with some studies showing an association of a better prognosis among the EBV-positive subgroup with others showing no difference [2,9].

The treatment of PbL is not clearly defined. Without any chemotherapy, the median survival is about three months [15]. The overall response rate with chemotherapy has shown to be about 77% with a median survival of 14 months and five-year survival of 31% [8]. Given the poor response to CHOP and CHOP-related regimens in these patients, the National Comprehensive Cancer Network (NCCN) guidelines favor the more intensive regimen - EPOCH (etoposide, prednisone, vincristine, cyclophosphamide, and doxorubicin hydrochloride), hyper-CVAD (cyclophosphamide, vincristine, adriamycin, and dexamethasone), and CODOX/M-IVAC (cyclophosphamide, vincristine, doxorubicin, high-dose methotrexate, and high-dose cytarabine). However, these intensive regimens have not shown any statistically significant survival benefit over CHOP/CHOP-related regimens [8].

Bortezomib has been shown to have some benefits in patients with PbL. This is thought to be due to the similar characteristics that PbL shares with plasmablastic plasma cell myeloma [16]. It has been used either as a single agent or in a combination with the CHOP/ EPOCH regimen [9].

## Conclusions

Plasmablastic lymphoma is an aggressive B-cell lymphoma with a guarded prognosis. It most commonly presents with extranodal involvement. Although rare, extra-nodal spread to the GI tract can be a presenting feature, and it must be distinguished from other lymphomas with plasmablastic features. Given the rarity of this disease and its aggressive course, a timely and accurate diagnosis of PbL is extremely crucial in determining the prognosis and tailoring the management of the disease.
